# Wenyang Huazhuo Tongluo formula alleviates pulmonary vascular injury and downregulates HIF-1α in bleomycin-induced systemic sclerosis mouse model

**DOI:** 10.1186/s12906-022-03651-9

**Published:** 2022-06-22

**Authors:** Kai Li, Qian Wang, Qin Lv, Kelei Guo, Li Han, Peipei Duan, Yongzheng Deng, Hua Bian

**Affiliations:** 1 Zhang Zhongjing College of Chinese Medicine, Nanyang Institute of Technology, Changjiang Road 80, Nanyang, 473004 Henan China; 2 Henan Key Laboratory of Zhang Zhongjing Formulae and Herbs for Immunoregulation, Nanyang Institute of Technology, Nanyang, Henan China; 3 Nanyang Medical College, Nanyang, Henan China; 4 Department of Rheumatology, The Second Affiliated Hospital of Henan University of Traditional Chinese Medicine, Zhengzhou, Henan China

**Keywords:** Wenyang Huazhuo Tongluo Formula, SSc mouse model, Pulmonary vascular injury, HIF-1α

## Abstract

**Background:**

Vascular damage, autoimmune abnormalities, and fibrosis are the three pathological features of systemic sclerosis (SSc).However, pulmonary vascular damage is the main factor affecting the progression and prognosis of SSc. The main purpose of this study was to explore the molecular mechanism of Wenyang Huazhuo Tongluo Formula in alleviating pulmonary vascular injury in bleomycin-induced SSc mouse model.

**Methods:**

Masson staining and H&E staining were used to analyze the degree of pulmonary vascular fibrosis and the infiltration of leukocyte cells in lung tissue ofbleomycin-induced SSc mouse models treated with saline (BLM group), Wenyang Huazhuo Tongluo Formula (WYHZTL group) and HIF-1α inhibitor KC7F2 (KC7F2 group). Blood vessel exudation was determined by analyzing the cell number and albumin concentration in bronchoalveolar lavage fluid using a cell counter and ELISA assay, respectively. The degree of vascular injury was assessed by measuring the expression levels of vWF, E-selectin, ICAM-1, VCAM-1, VE-cadherin and claudin-5 in serum and pulmonary vascular endothelial cells using ELISA and immunofluorescence staining. Finally, the effect of Wenyang Huazhuo Tongluo Formula on the expression of HIF-1α was detected using immunofluorescence staining.

**Results:**

Wenyang Huazhuo Tongluo Formula and KC7F2 significantly inhibited bleomycin-induced pulmonary vascular fibrosis and the level of perivascular inflammatory cell infiltration. The number of cells and the concentration of albumin were significantly reduced in the bronchoalveolar lavage fluid of the WYHZTL group and KC7F2 group compared with the BLM group. In addition, treatment with Wenyang Huazhuo Tongluo Formula and KC7F2 significantly downregulated the expression levels of vWF, E-selectin, ICAM-1, VCAM-1 and HIF-1α, but upregulated the expression of VE-cadherin and claudin-5 in serum and pulmonary vascular endothelial cells, compared with treatment with saline.

**Conclusions:**

This study reveals that Wenyang Huazhuo Tongluo Formula plays a new role in the treatment of SSc by alleviating pulmonary vascular damage. Furthermore, we found that Wenyang Huazhuo Tongluo Formula alleviates pulmonary vascular injury and inhibits HIF-1α expression.

**Supplementary Information:**

The online version contains supplementary material available at 10.1186/s12906-022-03651-9.

## Background

Systemic sclerosis (SSc) is an autoimmune disease characterized by vascular damage, autoimmune abnormalities, and progressive fibrosis of multiple organs [1]. Since skin and lungs are the main organs damaged by SSc, pulmonary vascular injury and pulmonary fibrosis are the main factors affecting the progression and prognosis of SSc. More than 70% of SSc patients also have pulmonary diseases, mainly interstitial lung disease and pulmonary hypertension. These two pulmonary diseases account for 60% of SSc-related deaths [2]. Early pathological changes such as microvascular injury and alveolar inflammation have been implicated in the pathogenesis of SSc-related pulmonary diseases. The inflammation and autoimmune response induced by microvascular injury directly or indirectly stimulate the activation of fibroblasts, leading to the occurrence of fibrosis [3]. The occurrence of pulmonary microvascular endothelial cell injury has been attributed to hypoxia, inflammation, hyperphagy and endothelial-mesenchymal transition (EndoMT) [4].

Traditional Chinese medicine has potential in the treatment of SSc. For example, Tripterygium wilfordii significantly alleviates the forced vital capacity of patients with SSc-related interstitial lung disease, in a similar manner to cyclophosphamide, but its side effects, such as the inhibition of white blood cells, are weaker than cyclophosphamide [5]. In addition, recent studies have found that Tanshinone IIA, an extract derived from Salvia miltiorrhiza, can significantly inhibit SSc-induced skin and lung fibrosis caused by collagen deposition, reverse the bleomycin-induced EndoMT by inhibiting the Akt/mTOR/p70S6K pathway in vivo and in vitro, and alleviate SSc-induced vessels damage [6]. Geniposide derived from gardenia can inhibit bleomycin-induced EndoMT of endothelial cells through the mTOR signaling pathway, thereby improving SSc fibrosis in vivo and in vitro [7]. Most studies on the use of traditional chinese medicine (TCM) in treating SSc have mainly focused on skin fibrosis, withvery few studies exploring their effect on pulmonary vascular injury.

Wenyang Huazhuo Tongluo Formula was invented by our team and has been widely used in the clinical treatment of SSc. Our previous studies have shown that Wenyang Huazhuo Tongluo Formula regulated immune imbalance and exerts anti-fibrosis effects in the treatment of SSc. Wenyang Huazhuo Tongluo Formula exerts it anti-fibrosis effects by regulating the fibroblast cell cycle, inhibiting its proliferation, and reducing collagen synthesis by inhibiting the Wnt/β-catenin signaling pathway [8–10]. Wenyang Huazhuo Tongluo Formula can also inhibit the expression of type I and III collagen by inhibiting the TGF-β1/Smad signaling pathway in SSc fibroblasts. In addition, Wenyang Huazhuo Tongluo Formula can also improve fibrosis by upregulating MMP-9, inhibiting the expression of TIMP-1, and redressing the imbalance of MMP-9/TIMP-1 [11]. Wenyang Huazhuo Tongluo Formula regulates immune imbalance by alleviating the Th17/Treg cells imbalance in SSc [12]. In clinical treatment, we found that Wenyang Huazhuo Tongluo Formula could significantly ameliorate the hypoxic symptoms of SSc through unknown mechanisms.

The main purpose of this study was to explore the therapeutic effects ofWenyang Huazhuo Tongluo Formula against pulmonary vascular injury during SSc treatment. In addition, we also demonstrated that Wenyang Huazhuo Tongluo Formula alleviates pulmonary vascular injury and regulates HIF-1α expression.

## Methods

### Construction of bleomycin-induced SSc mouse model and measurement of skin thickness

Male C57/BL6N mice (purchased from Beijing Vital River Laboratory Animal Technology Co., Ltd.) aged 8 weeks and weighing 17-20 g were used to construct a bleomycin-induced SSc mouse model as previously described [8]. Briefy, 100 ul of bleomycin (200 ug/ml) was subcutaneous injected to the restricted area of the upper back each day, which lasted for 3 weeks. PBS was used as a negative control during the bleomycin treatment. The study protocol was approved by the Ethics Committee of Nanyang Institute of Technology. A skin caliper was used to measure the skin thickness of the lesions of each group of mice as previously described [8]. All animal experiment procedures were performed in compliance with the ARRIVE guidelines and other relevant guidelines and regulations.

### Drug preparation and experiment grouping

The ingredients and dosage of Wenyang Huazhuo Tongluo Formula are presented in Table 1. Wenyang Huazhuo Tongluo Formula was prepared at the Affiliated Hospital of Nanyang Institute of Technology, as previously described [8]. Briefy, all of the ingredients of WYHZTL were prepared as crude slices and boiled twice (90 min per time) with ultrapure water as the doctor’s directions. The water extracts were combined, filtered and evaporated under reduced pressure to a final concentration of 1.5 g/mL based on the equivalent amount of the crude drugs. Fifty C57/BL6 mice were randomly divided into 5 groups of 10 mice each. The 5 groups included a no treatment group (labeled as the Normal group), a group of mice injected subcutaneously with 100ul PBS solution (labeled as the PBS group), and three groups of mice used to construct the bleomycin-induced SSc mouse model (labeled as BLM group). The drug treatment plan is: normal group mice, PBS group mice and one of the BLM group received daily intragastric administration of normal saline (1 ml/day), another group receivedintragastric administration of Wenyang Huazhuo Tongluo Formula (47 g/kg/day, labeled as WYHZTL group), and the last group received subperitoneal injection of KC7L2 (dissolved in PBS solution, 10 mg/kg/day, labeled as KC7L2 Group). All treatmentstarted concurrently with bleomycin and lasted for 4 weeks, and then the mice were anesthetized with an intraperitoneal injection of a mixture of ketamine (150 mg/kg) and acepromazine (15 mg/kg). Finally, mice were euthanized by cervical dislocation under anesthesia. Bronchoalveolar lavage fluid (BALF), serum, skin and lung tissue were then collected as previously described [13]. The serum was stored at -80 °C, while the skin and lung tissue were immediately fixed in formalin solution.

**Table 1:** The herbal ingredients and dosage of Wenyang Huazhuo Tongluo Formula

number	herbal ingredients	dosage (g)
1	Radix Astragali membranacei	30
2	Radix Codonopsitis pilosulae	15
3	Radix Dioscoreae oppositae	12
4	Herba Epimedii	12
5	Radix Rehmanniae praeparata	15
6	Ramulus Cinnamomi cassiae	9
7	Herba Glechomae longitubae	15
8	Semen Sinapis lbae	9
9	Capparis zeylanica Linn	15
10	Fasciculus vascularis Lufae	9

### Detection of the cell number in BALF

The cell number and albumin concentration in BALF was determined immediately after collection. The BALF was centrifuged at 1500 rpm/min for 10 min,and the cell pellet used for determination of cell number while the supernatant was retained for ELISA assays.The cell pellet was resuspended in 200ul PBS solution, and then detected with LUNA-II cell counter (Logos Biosystems, Korea).

### Enzyme-linked immunosorbent assay (ELISA)

The concentrations of albumin in BALF as well as vWF, SELE, ICAM-1 and VCAM-1 in the serum were determined using ELISA assays. The ELISA kits were purchased from Cusabio Biotech, and the assays were carried out in accordance with the kit instructions.

### Hematoxylin–eosin (H&E) staining and analysis of lung injury 

The skin and lung tissues were fixed in formalin, and then embedded in paraffin for sectioning. The tissue sections were stained with the H&E staining kit (Solarbio Technology Co., LTD, Beijing) according to the manufacturer’s instructions. The stained lung tissue sections were used to assess lung injury under a microscope according to the following scoring criteria: ①Edema: 1 point represents non-existent, 2 point represents mild (10% alveolar involvement), 3 point represents moderate (10–50% alveolar involvement) or 4 point represents severe (50% alveolar involvement); ②Inflammation: 1 point represents none, 2 point represents mild (10 inflammatory cells/each high power field), 3 point represents moderate (10–50 inflammatory cells/each high power field), or 4 point represents severe (50 inflammatory cells/each high power field); ③Hyaline membrane: 1 point represents absence, 2 point represents presence. The assessment of lung injury was carried out by three pathologists in accordance with the double-blind principle.

### Masson staining and analysis of lung fibrosis

Lung tissue sections were also stained with masson stain according to the manufacturer’s instructions. Collagen appears blue after masson staining and can be used to assess the degree of lung fibrosis. The degree of pulmonary fibrosis is measured using the Ashcroft score, which uses the following criteria: 0 represent normal lung tissue; 1 represents slight alveolar septum or bronchial wall thickening; 2 to 3 represent moderate alveolar wall or bronchial wall thickening, but no lung structure damage; 4 to 5 represent fibrous tissue hyperplasia with obvious lung structural damage or fibrous tissue mass formation; 6 to 7 represent severe lung structural damage and large-scale fibrous tissue formation, honeycomb lung; 8 represent observation of fibrous tissue in the full view under observation. The above results were analyzed separately by three pathologists in accordance with the double-blind principle.

### Immunofluorescence staining

Immunofluorescence staining was used to detect the expression levels of vWF, SELE, ICAM-1, VCAM-1, HIF-1α, VE-cadherin and claudin-5 in pulmonary vascular tissues, as previously described [14]. The nuclei were stained using DAPI staining, while vWF, SELE, ICAM-1, VCAM-1, HIF-1α, VE-cadherin and claudin-5 proteins were labeled with green fluorescence. The antibodies against vWF, SELE, ICAM-1, VCAM-1, HIF-1α, VE-cadherin and claudin-5 were purchased form Affinity Biosciences Company. According to the previous study, the quantitative analysis of immunofluorescence staining was performed using image J [15].

### Statistical analysis

The experimental results were expressed as mean ± standard deviation. Graphpad Prism 7.0 was used for statistical analysis. Differences among groups were analyzed using one-way ANOVA followed by Tukey’s post-test. P < 0.05 was considered to be statistically significant.

## Results

### Wenyang Huazhuo Tongluo formula significantly alleviates skin and lung pathological changes in the bleomycin-induced SSc mouse model

**Fig. 1:**
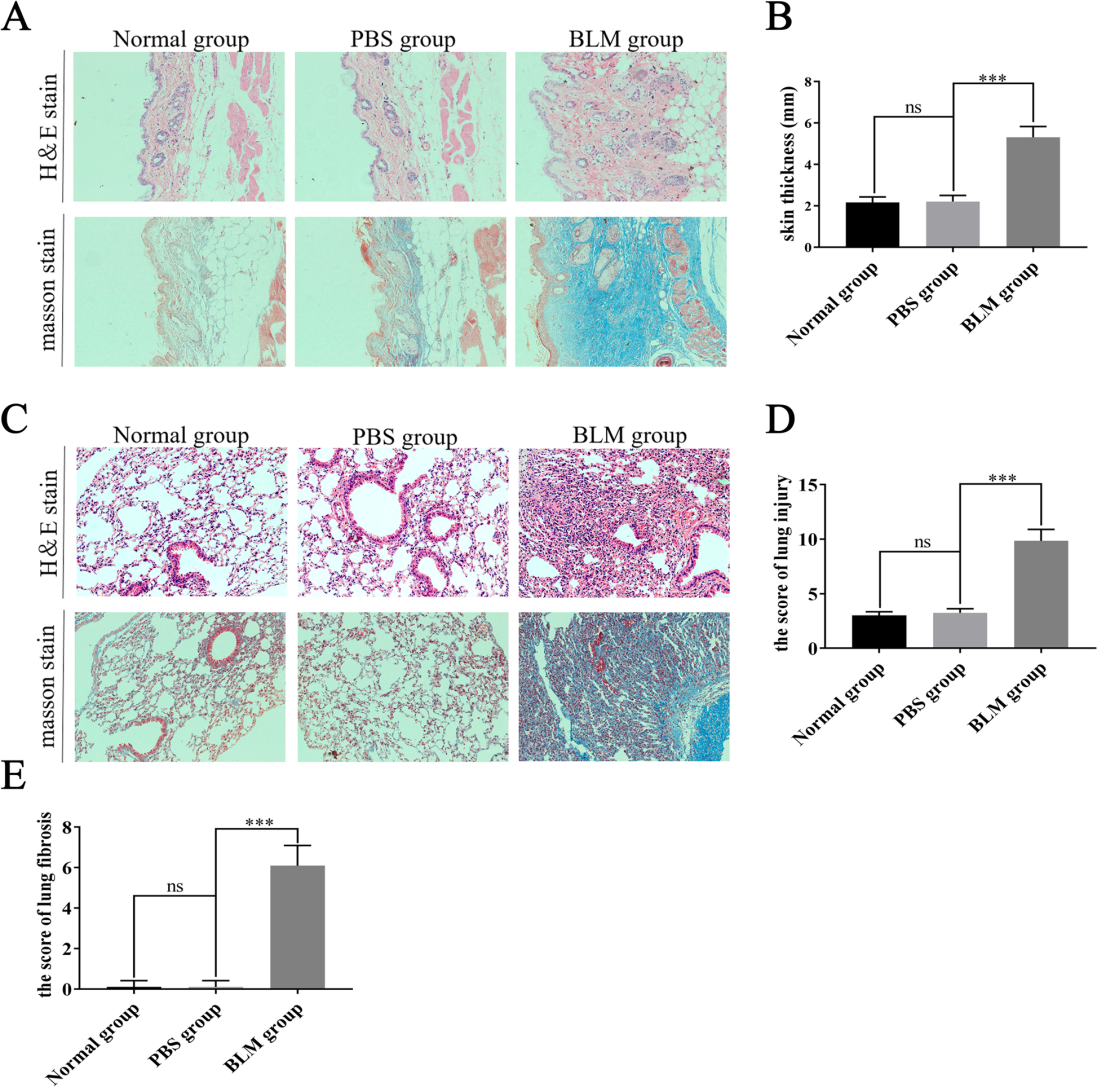
The bleomycin-induced SSc mouse model was successfully constructed. The pathological changes (**A**) and thickness (**B**) of different groups of skin tissues were detected by H&E staining, masson staining and a skin caliper. The pathological changes (**C**), the score of lung injury (**D**) and the score of fibrosis (**E**) were analyzed by H&E staining and masson staining. The mean values ± SD was shown for each bar. *** (*P* < 0.001) represents significance, ns represents no significance. Original magnification: × 20. BLM: Bleomycin, WYHZTL: Wenyang Huazhuo Tongluo formula

The results of H&E staining and masson staining showed a significant increase in the skin thickness and the collagen deposition of the BLM group compared with the PBS group(Fig. 1A-B). In addition, quantitative evaluation of pathological changes in lung tissues showed that lung injury and fibrosis in the BLM group were more serious and mainly manifested as pulmonary edema, inflammatory cell infiltration, and thickening of alveolar septum caused by collagen deposition, and even the disappearance of alveolar structure, compared with the PBS group **(**Fig. 1C-E**)**. These results indicated that the bleomycin-induced SSc mouse model had been successfully constructed.

### Wenyang Huazhuo Tongluo formula alleviates the structural abnormalities of the pulmonary vessels in the bleomycin-induced SSc mouse model

**Fig. 2:**
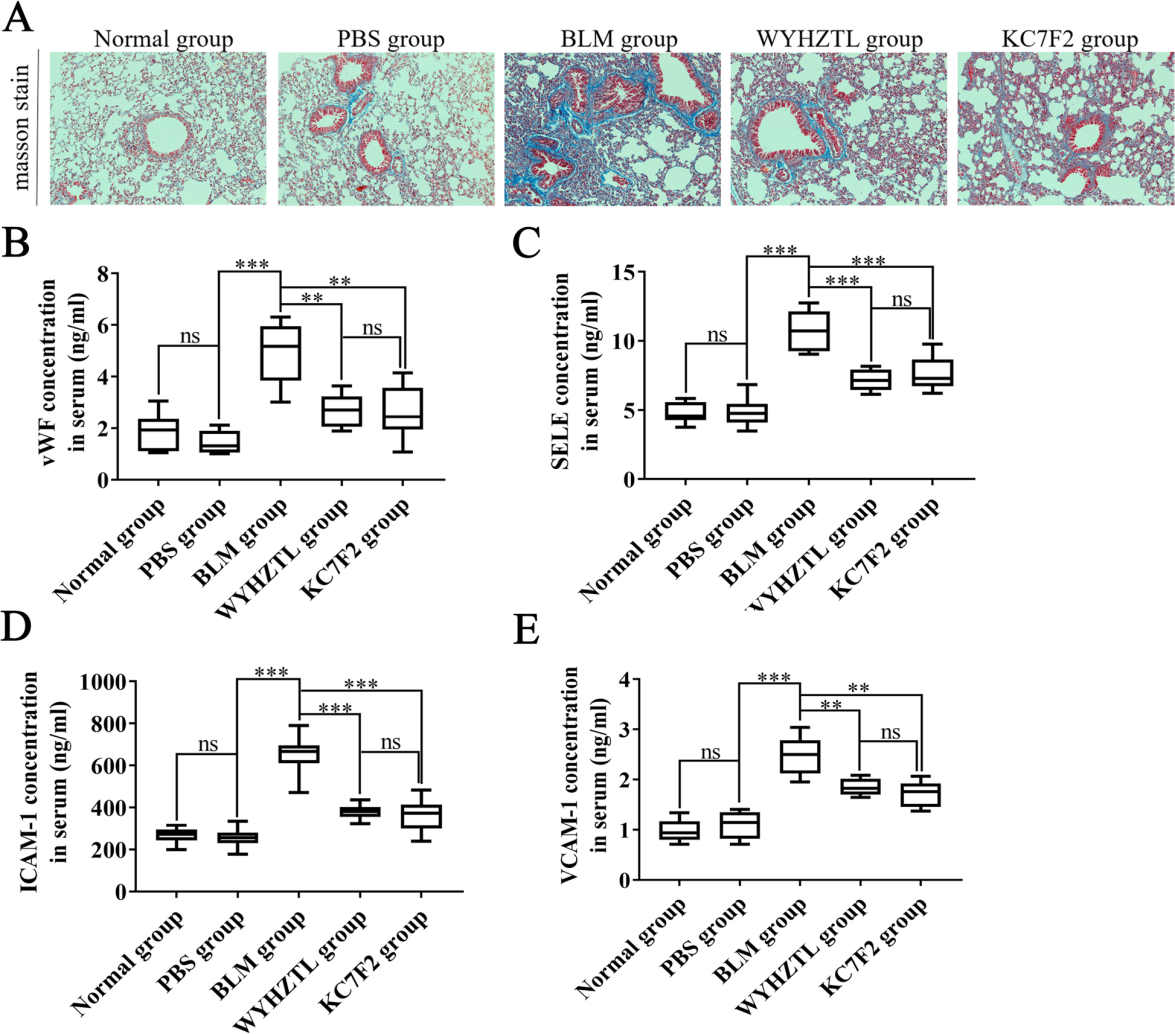
Wenyang Huazhuo Tongluo Formula can alleviate the structural abnormalities of the pulmonary vessels in the bleomycin-induced SSc mouse model. **A** The masson stain of lung tissues showed that Wenyang Huazhuo Tongluo Formula and KC7F2 can significantly alleviate the pulmonary vascular fibrosis in SSc mouse models. In additional, Wenyang Huazhuo Tongluo Formula and KC7F2 can significantly reduce the concentration of vWF (**B**), SELE (**C**), ICAM-1 (**D**) and VCAM-1 (**E**) in serum, which were detected by ELISA. The mean values ± SDwas shown for each bar. * (*P* < 0.05) or ** (*P* < 0.01) or *** (*P* < 0.001) represents significance, ns represents no significance. Original magnification: × 20. BLM: Bleomycin, WYHZTL: Wenyang Huazhuo Tongluo formula

Vascular injury is an early event in the pathogenesis of SSc that appears before the development of fibrosis and immune abnormalities [16]. In this study, we assessed vascular fibrosis in the lung tissue of the SSc mouse model using masson staining, and found that the pulmonary vessels of the mice in the BLM group exhibited significant fibrosis and reduction in thediameter of the vessels compared with the PBS group (Fig. 2A). Treatment of mice with Wenyang Huazhuo Tongluo Formula and KC7F2 significantly alleviated the pulmonary vascular fibrosis observed in the SSc mouse models (Fig. 2A).

SSc-induced pulmonary vascular injury is characterized by the gradual loss of capillaries caused by the necrosis of vascular endothelial cells, which ultimately leads to tissue hypoxia and activation of dermal fibroblasts.Necrosis of endothelial cells can be evaluated using specific protein markers in serum, such as von Willebrand factor, vascular cell adhesion molecule-1 (VCAM-1), intercellular adhesion molecule-1 (ICAM-1), and E-selectin (SELE) [17–19]. In this study, the concentrations of vWF, SELE, ICAM-1 and VCAM-1 in the serum of the BLM group mice, were significantly higher than those in the PBS group (Fig. 2B-E). These resultswere a reflection of serious vascular damage in the bleomycin-induced SSc mouse model. More importantly, Wenyang Huazhuo Tongluo Formula and KC7F2 significantly reduced the concentrations of vWF, SELE, ICAM-1 and VCAM-1 in serum (Fig. 2B-E), suggesting that Wenyang Huazhuo Tongluo Formula and KC7F2 can protect vascular endothelial cells and alleviate vessel damage in SSc.

### Wenyang Huazhuo Tongluo formula alleviatespulmonary vascular leakage in bleomycin-induced SSc mouse model

**Fig. 3:**
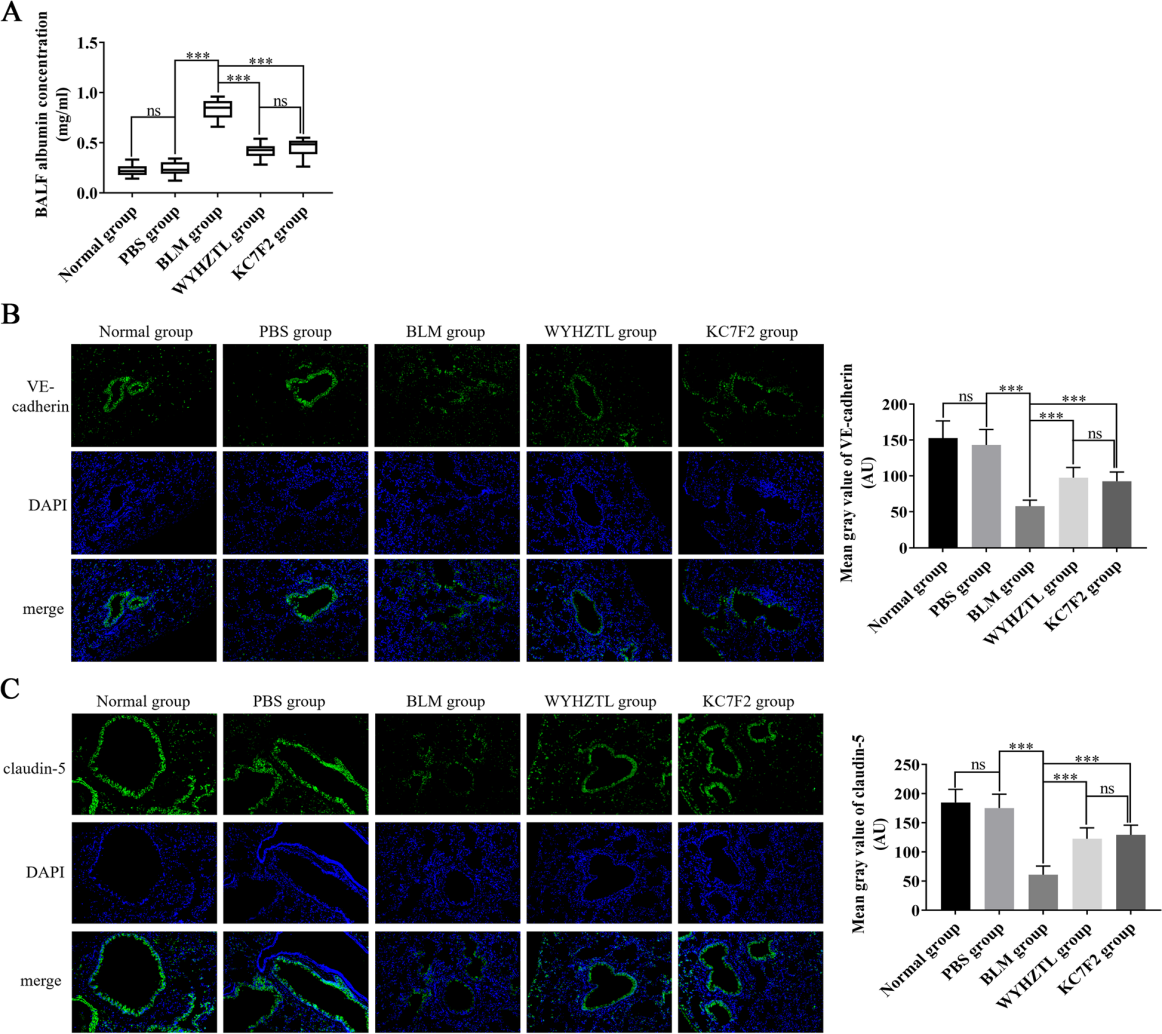
Wenyang Huazhuo Tongluo Formula can alleviate the pulmonary vascular leakage in bleomycin-induced SSc mouse model. **A** The concentration of albumin in BALF of different group mice was detected by ELISA assay. Then, the expression of VE-cadherin (**B**) and claudin-5 (**C**) were detected by immunofluorescence staining, in which VE-cadherin and claudin-5 were labeled with green fluorescence, while the nuclear was stained by DAPI with blue fluorescence. The mean values ± SD was shown for each bar. * (*P* < 0.05) or ** (*P* < 0.01) or *** (*P* < 0.001) represents significance, ns represents no significance. Original magnification: × 20. BLM: Bleomycin, WYHZTL: Wenyang Huazhuo Tongluo formula

In SSc, damage factors such as TGF-β, vascular endothelial growth factor, hypoxic stress response, and reactive oxygen species act on vascular endothelial cells, and may cause abnormalities in the function and structure of endothelial cells.These abnormalities lead to the downregulation of tight junctions (claudin, occludin and JAMs family proteins), adhesion junctions (VE-cadherin and catenin proteins), and gap junctions between cells, which in turn destroys the integrity of vessels and finally causes vascular leakage [20, 21]. In this study, we tested the albumin concentration in the BALF of mice in different groups using ELISA. We found that the albumin concentration in the BALF of the BLM group was significantly higherthan the PBS group, and that Wenyang Huazhuo Tongluo Formula and KC7F2 significantly reversed the upregulation of albumin concentration (Fig. 3A). These results indicated that bleomycin can induce increased vascular permeability resulting in albumin exudation, while Wenyang Huazhuo Tongluo Formula and KC7F2 can alleviate the pulmonary vascular leakage in SSc. We further explored the molecular mechanisms of Wenyang Huazhuo Tongluo Formula and KC7F2 action in alleviating pulmonary vascular leakagethrough immunofluorescence staining of VE-cadherin and claudin-5.We found that the expression levels of VE-cadherin and claudin-5 protein in the pulmonary vascular endothelial cells of BLM group mice were significantly reduced, while Wenyang Huazhuo Tongluo Formula and KC7F2 upregulated their expression levels in pulmonary vascular endothelial cells (Fig. 3B, C). These results indicated that Wenyang Huazhuo Tongluo Formula and KC7F2 can alleviate pulmonary vascular leakage by regulating the expression levels of VE-cadherin and claudin-5.

### Wenyang Huazhuo Tongluo formula alleviates the functional abnormalities of the pulmonary vessels in the bleomycin-induced SSc mouse model

**Fig. 4:**
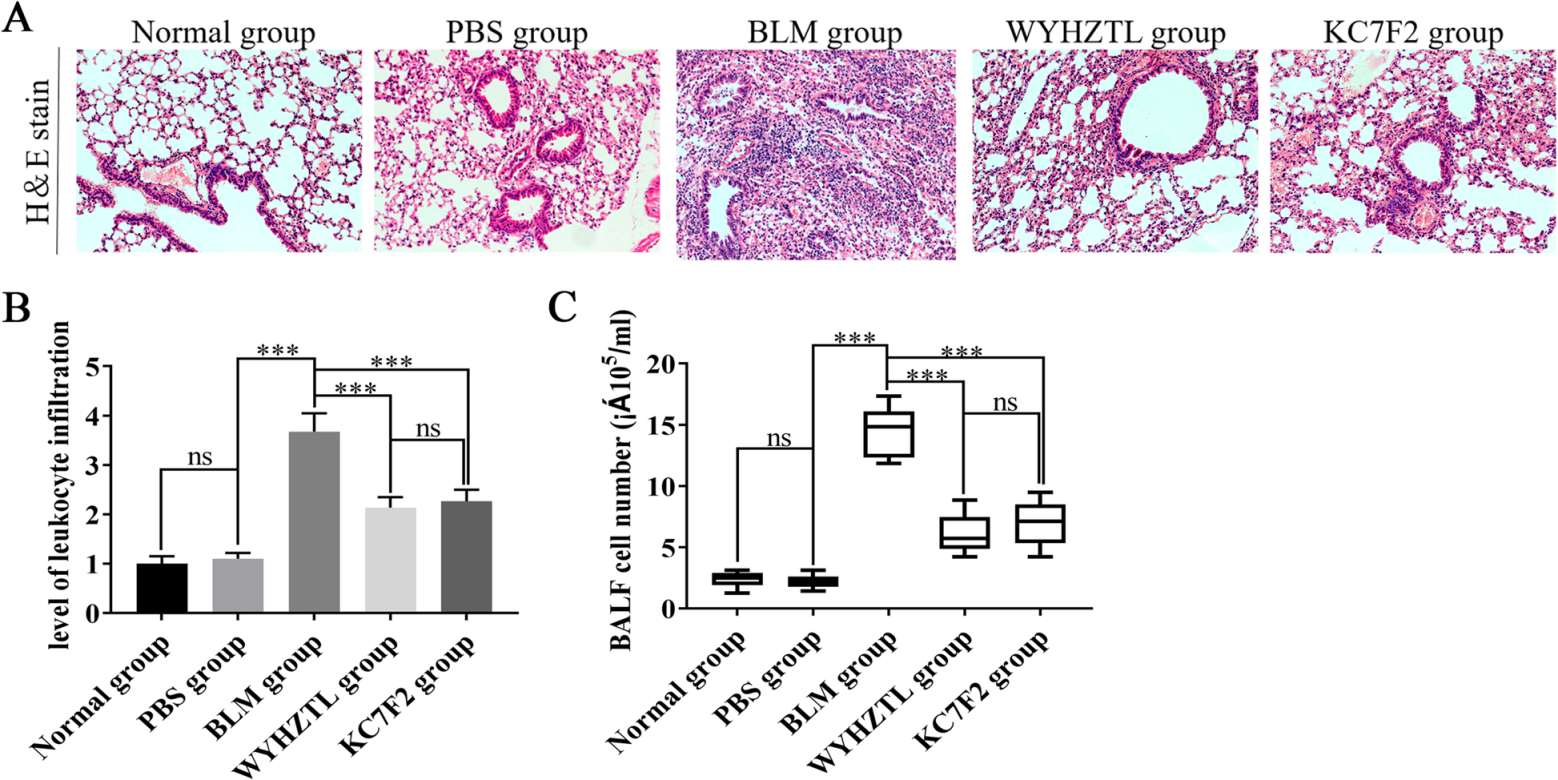
Wenyang Huazhuo Tongluo Formula and KC7F2 could significantly inhibit inflammatory cell infiltrationin the lungs of bleomycin-induced SSc mouse model. **A**, **B**) The levels of leukocyte infiltration in lungs of different group mice were observed by H&E stain. (**C**) The number of cells in BALF of different group mice were detected by cell counter. The mean values ± SD was shown for each bar. * (*P* < 0.05) or ** (*P* < 0.01) or *** (*P* < 0.001) represents significance, ns represents no significance. Original magnification: × 20. BLM: Bleomycin, WYHZTL: Wenyang Huazhuo Tongluo formula

During the occurrence and development of SSc, the activation of endothelial cells is the main manifestation of vascular endothelial cell dysfunction, which is mainly characterized by increased expression of adhesion molecules on the surface of endothelial cells [16]. Cell adhesion molecules such as vWF, ICAM-1, VCAM-1 and SELE located on the surface of vascular endothelial cells are necessary for the adhesion of platelets and leukocytes [22]. In this study, H&E staining was used to investigate the infiltration of lungs by inflammatory cells, especially around the pulmonary vessels. The results showed that there was a large number of infiltrating inflammatory cells around the pulmonary vessels in the BLM group, a condition that was significantly inhibited by treatment with Wenyang Huazhuo Tongluo Formula and KC7F2 (Fig. 4A, B). We also analyzed the number of cells in the BALF, and found that the results were consistent with H&E staining (Fig. 4C).

**Fig. 5:**
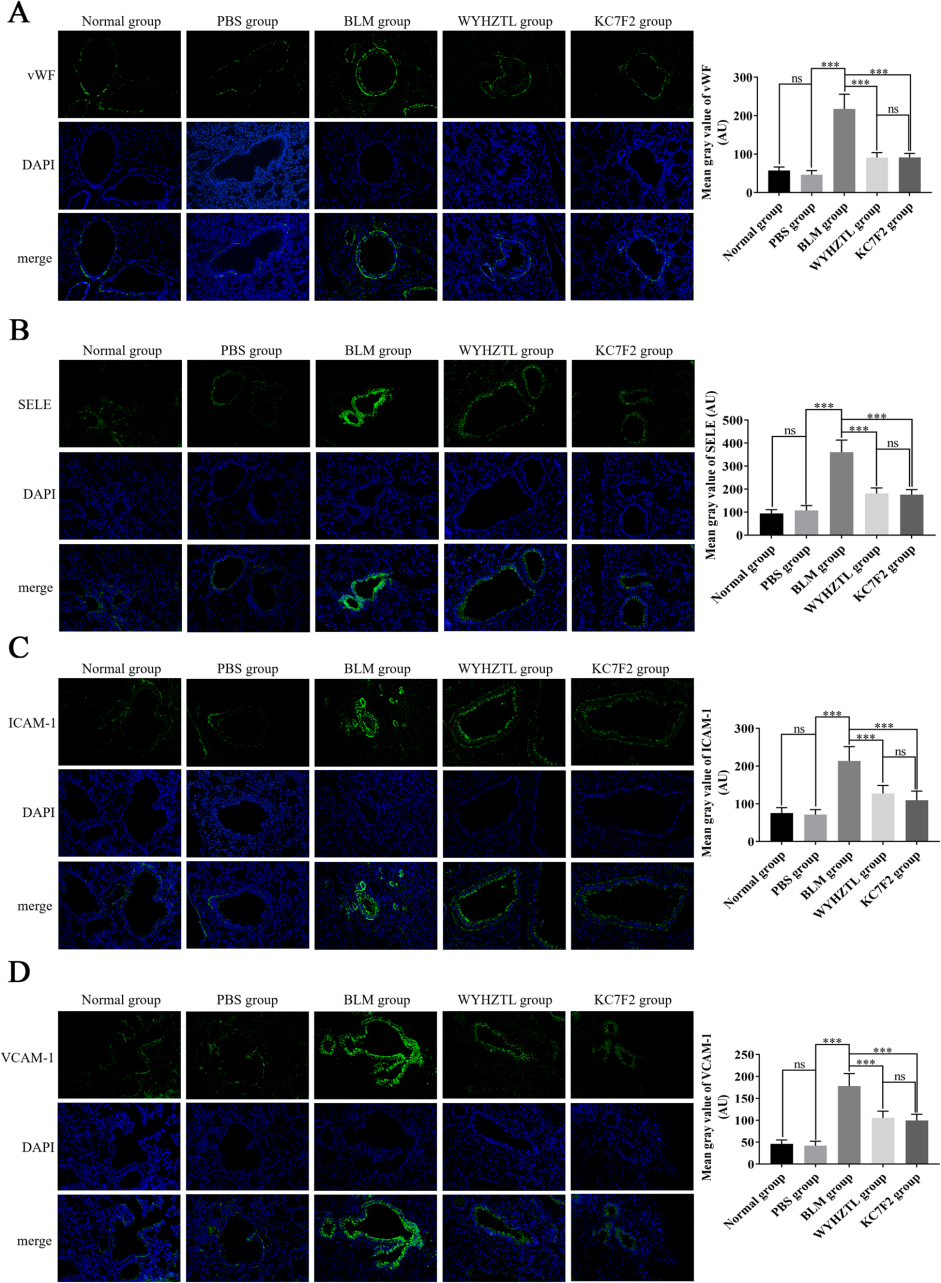
Wenyang Huazhuo Tongluo Formula and KC7F2 can significantly inhibit the expression of vWF, SELE, ICAM-1 and VCAM-1 in bleomycin-induced SSc mouse model. The effect of Wenyang Huazhuo Tongluo Formula and KC7F2 on the expression of vWF (**A**), SELE (**B**), ICAM-1 (**C**) and VCAM-1 (**D**) was observed by immunofluorescence staining. compared with the PBS group, bleomycin can significantly upregulate the expression levels of vWF, SELE, ICAM-1 and VCAM-1 in endothelial cells, while Wenyang Huazhuo Tongluo Formula and KC7F2 can significantly reverse the bleomycin-induced upregulation of vWF, SELE, ICAM-1 and VCAM-1. The mean values ± SD was shown for each bar. * (*P* < 0.05) or ** (*P* < 0.01) or *** (*P* < 0.001) represents significance, ns represents no significance. Original magnification: × 20. BLM: Bleomycin, WYHZTL: Wenyang Huazhuo Tongluo formula

Furthermore, we analyzed endothelial cell adhesion molecules which were closely related to inflammatory cell exudation. As expected, bleomycin significantly upregulated the expression levels of vWF, SELE, ICAM-1 and VCAM-1 in endothelial cells compared with PBS, while Wenyang Huazhuo Tongluo Formula and KC7F2 significantly reversed the bleomycin-induced upregulation of vWF, SELE, ICAM-1 and VCAM-1 (Fig. 5A-D). These findings show that Wenyang Huazhuo Tongluo Formula and KC7F2 can inhibit the activation of pulmonary vascular endothelial cells and alleviate SSc pulmonary vascular dysfunction.

### Wenyang Huazhuo Tongluo formula alleviates pulmonary vascular endothelial cell injury in bleomycin-induced SSc mouse model and regulates HIF-1α

**Fig. 6:**
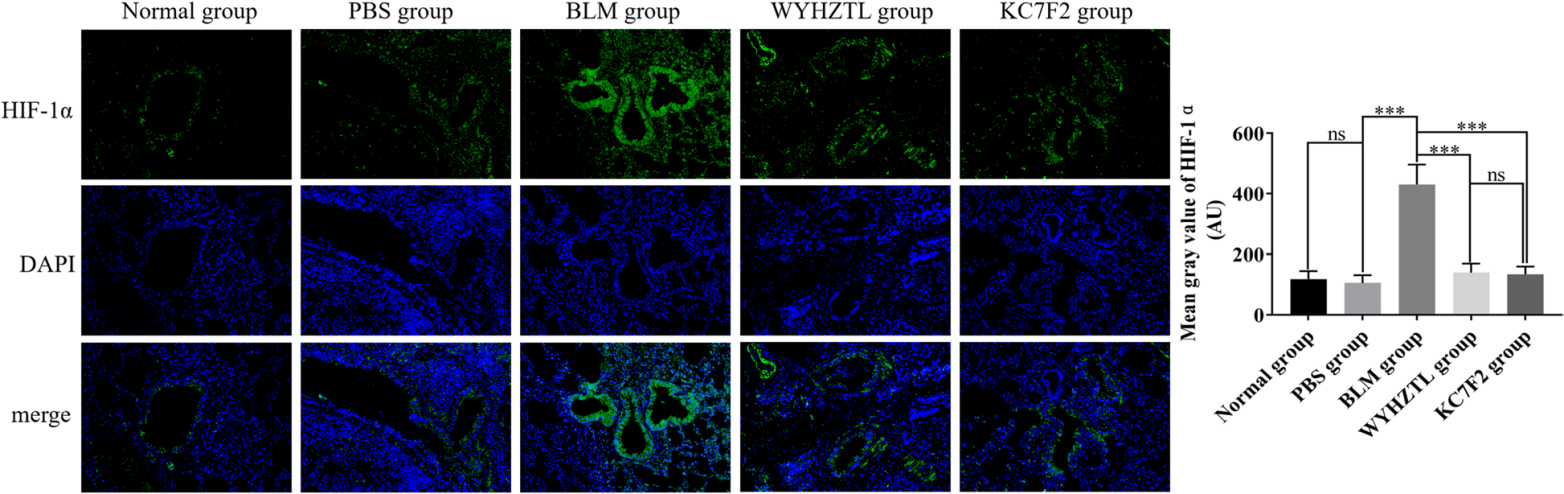
Wenyang Huazhuo Tongluo Formula and KC7F2 can significantly inhibit the expression of HIF-1α in bleomycin-induced SSc mouse model. The effect of Wenyang Huazhuo Tongluo Formula and KC7F2 on the expression of HIF-1α was observed by immunofluorescence staining, in which HIF-1α were labeled with green fluorescence, while the nuclear was stained by DAPI with blue fluorescence. The mean values ± SD was shown for each bar. * (*P* < 0.05) or ** (*P* < 0.01) or *** (*P* < 0.001) represents significance, ns represents no significance. Original magnification: × 20. BLM: Bleomycin, WYHZTL: Wenyang Huazhuo Tongluo formula

KC7F2 is widely used as the inhibitor of HIF-1α [23–25]. In this study, we demonstrated that KC7F2 can significantly alleviate pulmonary vascular injury, an indication that inhibiting HIF-1α is one of the effective measures to alleviate pulmonary vascular injury. Therefore, we explored the effects of Wenyang Huazhuo Tongluo Formula on HIF-1α in the bleomycin-induced SSc mouse model using immunofluorescence. The results showed that the expression levels of HIF-1α in the pulmonary vascular endothelial cells of the BLM group were significantly higher than those in the PBS group, and that Wenyang Huazhuo Tongluo Formula significantly reversed the upregulation of HIF-1α in BLM group (Fig. 6). These results indicated that the Wenyang Huazhuo Tongluo Formula may alleviate SSc pulmonary vascular injury by regulating the expression of HIF-1α.

## Discussion

The correlation between SSc vascular injury and hypoxia is attributed to the loss of capillaries and the remodeling of arterioles during the occurrence and development of SSc, which results in severe hypoxia of tissue cells, including vascular endothelial cells. Hypoxia further aggravates vascular damage by directly causing dysfunction of vascular endothelial cell and inducing cell necrosis through oxidative stress and other mechanisms, which forms a vicious circle [26]. Therefore, breaking the vicious circle between vascular injury and hypoxia can be one of the strategies for the treatment of SSc.

The SSc-induced vascular injury includes two aspects: structural abnormalities and dysfunction of vascular endothelial cell [27, 28]. The mechanisms resulting in structural abnormalities and dysfunction of vascular endothelial cell are closely related to hypoxia [29]. vWF, VCAM-1, ICAM-1 and SELE are mainly synthesized in vascular endothelial cells and distributed in cells or between cells. However, damage factors can induce endothelial cell necrosis, leading to the release of these proteins from endothelial cells into the blood, resulting in an increased concentration in the serum. Many studies have reported a significant increase in the serum levels of vWF, SELE, ICAM-1 and VCAM-1 in SSc patients, which is closely related to the disease progression [30–32]. In this study,we demonstrated that Wenyang Huazhuo Tongluo Formula can significantly reduce the concentrations of vWF, SELE, ICAM-1 and VCAM-1 in serum, suggesting that Wenyang Huazhuo Tongluo Formula can protect vascular endothelial cells and alleviate SSc-induced structural abnormalities.

HIF-1α is the core factor in hypoxic stress response [33] that is up-regulated in SSc, especially in SSc patients with vascular lesions [34]. Under hypoxic conditions, HIF-1α plays a key role in extensive vascular remodeling, ultimately leading to the occurrence of pulmonary hypertension, which is one of the important causes of death in SSc patients [35]. The results of a recent study showed that HIF-1α gene polymorphism was not associated with susceptibility to SSc, but was significantly associated with the severity of PAH, suggesting that HIF-1α may be involved in vascular damage in SSc [36]. In the present study, we observed the upregulation of HIF-1α in the SSc mouse model. In addition, KC7F2 can significantly improve lung tissue fibrosis and vascular injury in SSc mouse model, suggesting that HIF-1α plays an important role in SSc-induced lung injury and may be a potential target for the treatment of SSc. In this study, we found for the first time that Wenyang Huazhuo Tongluo Formula has an effect similar to KC7F2 in improving lung tissue fibrosis and vascular injury in SSc mouse model.

Dysfunction of vascular endothelial cells is also one of the important pathogenesis of SSc [37]. Injury factors induce changes in the expression levels of cell adhesion molecules, chemokines, cytokines and growth factors in vascular endothelial cells, resulting in the activationand enhanced infiltration of inflammatory cells into the perivascular area. Microvascular thrombosis, which is also common in SSc, is closely related to the expression of the adhesion molecule vWF on the surface of endothelial cells. The intensity and nature of inflammation are largely regulated by cell adhesion molecules expressed on endothelial cells. Uncontrollable inflammation causes tissue damage and promotes fibrosis [38]. Therefore, inhibiting the activation of SSc vascular endothelial cells is anotherstrategy for the treatment of SSc. Studies have also confirmed that HIF-1α can regulate the expression levels of vWF, VCAM-1, ICAM-1, and SELE [39–41]. In our study, we found that Wenyang Huazhuo Tongluo Formula inhibited the expression levels of HIF-1α, vWF, VCAM-1, ICAM-1, and SELE in pulmonary vascular endothelial cells. Therefore, we speculated that Wenyang Huazhuo Tongluo Formula could downregulate vWF, VCAM-1, ICAM-1, and SELE by inhibiting the expression of HIF-1α, thereby alleviating SSc-induced pulmonary vascular injury.

## Conclusions

In summary, this study is part of a seriesof studies on the treatment of SSc using Wenyang Huazhuo Tongluo Formula. Our previous studies demonstrated that Wenyang Huazhuo Tongluo Formula has anti-fibrosis effects and regulates immune imbalance. In this study, we further demonstrated that Wenyang Huazhuo Tongluo Formula alleviates SSc-induced pulmonary vascular damage and inhibits HIF-1α. Our findings provide sufficient evidence to support the clinical application of Wenyang Huazhuo Tongluo Formula, and more importantly, a useful reference for the development of new drugs for SSc.

## Supplementary Information

**Additional file 1. **The original images of H&E staining, massonstaining and immunofluorescence staining.

## Data Availability

The data used to support the findings of this study are available from the corresponding author upon request.

## Abbreviations

SSc: Systemic Sclerosis
ROS: Reactive Oxygen Species
EndoMT: Endothelial-Mesenchymal Transition
TCM: Traditional Chinese Medicine
BALF: Bronchoalveolar Lavage Fluid
ELISA: Enzyme-Linked Immunosorbent Assay
H&E: Hematoxylin-Eosin
vWF: Von Willebrand Factor
VCAM-1: Vascular Cell Adhesion Molecule-1
ICAM-1: Intercellular Adhesion Molecule-1
SELE: E-selectin
HIF-1α: Hypoxia-Inducible Factors-1α

## References

[1] Jerjen R, Nikpour M, Krieg T, Denton CP, Saracino AM. Systemic sclerosis in adults. Part I: Clinical features and pathogenesis. J Am Acad Dermatol. 2022;S0190–9622(22)00190–6. 10.1016/j.jaad.2021.10.065 35131402

[2] Perelas A, Silver RM, Arrossi AV, Highland KB (2020). Systemic sclerosis-associated interstitial lung disease. Lancet Respir Med.

[3] Cutolo M, Soldano S, Smith V (2019). Pathophysiology of systemic sclerosis: current understanding and new insights. Expert Rev Clin Immunol.

[4] Bellando-Randone S, Matucci-Cerinic M (2019). Very early systemic sclerosis. Best Pract Res Clin Rheumatol.

[5] Yang L, Wang Q, Hou Y, Zhao J, Li M, Xu D, Zeng X (2020). The Chinese herb Tripterygium wilfordii Hook F for the treatment of systemic sclerosis-associated interstitial lung disease: data from a Chinese EUSTAR Center. Clin Rheumatol.

[6] Jiang Y, Hu F, Li Q, Shen C, Yang J, Li M (2019). Tanshinone IIA ameliorates the bleomycin-induced endothelial-to-mesenchymal transition via the Akt/mTOR/p70S6K pathway in a murine model of systemic sclerosis. Int Immunopharmacol.

[7] Qi Q, Mao Y, Tian Y, Zhu K, Cha X, Wu M, Zhou X (2017). Geniposide inhibited endothelial-mesenchymal transition via the mTOR signaling pathway in a bleomycin-induced scleroderma mouse model. Am J Transl Res.

[8] Wang Q, Zang W, Han L, Yang L, Ye S, Ouyang J, Zhang C, Bi Y, Zhang C, Bian H (2018). Wenyang Huazhuo Tongluo formula inhibits fibrosis via suppressing Wnt/beta-catenin signaling pathway in a Bleomycin-induced systemic sclerosis mouse model. Chin Med.

[9] Han L, Bian H, Ouyang J, Bi Y, Yang L, Ye S (2016). Wenyang Huazhuo Tongluo formula, a Chinese herbal decoction, improves skin fibrosis by promoting apoptosis and inhibiting proliferation through down-regulation of survivin and cyclin D1 in systemic sclerosis. BMC Complement Altern Med.

[10] Bian H, Fan YS, Lou LH, Mao BY, Shi JY (2009). Effect of Wenyang Huazhuo Tongluo recipe contained serum on proliferation and cell cycle of systemic sclerosis skin fibroblasts. Zhong Yao Cai.

[11] Bian H, Lv Q, Huang XZ, Hu JL, Yang L, Mao BY (2015). Effects of Wenyang Huazhuo Tongluo Recipe Containing Serum on Transforming Growth Factor beta1/ Smad Signaling Pathway of Skin Fibroblasts in Systemic Sclerosis. Zhongguo Zhong Xi Yi Jie He Za Zhi.

[12] Bian H, Yuan M (2015). Gao Z-m, Bi XD, Han L, Hu JL, Mao BY: **[Effect of Wenyang Huazhuo Tongluo Recipe on Peripheral Blood Thl7/Treg Cell Balance in Systemic Sclerosis Patients]**. Zhongguo Zhong Xi Yi Jie He Za Zhi.

[13] Zhang X, Chen J, Xue M, Tang Y, Xu J, Liu L, Huang Y, Yang Y, Qiu H, Guo F (2019). Overexpressing p130/E2F4 in mesenchymal stem cells facilitates the repair of injured alveolar epithelial cells in LPS-induced ARDS mice. Stem Cell Res Ther.

[14] Li K, Zhang J, Tian Y, He Y, Xu X, Pan W, Gao Y, Chen F, Wei L (2020). The Wnt/beta-catenin/VASP positive feedback loop drives cell proliferation and migration in breast cancer. Oncogene.

[15] Jensen EC (2013). Quantitative analysis of histological staining and fluorescence using ImageJ. Anat Rec (Hoboken).

[16] Pacholczak-Madej R, Kuszmiersz P, Bazan-Socha S, Kosalka-Wegiel J, Iwaniec T, Zareba L, Kielczewski S, Rams A, Walocha JA, Musial J (2020). Endothelial dysfunction in patients with systemic sclerosis. Postepy Dermatol Alergol.

[17] Utsunomiya A, Oyama N, Hasegawa M (2020). Potential biomarkers in systemic sclerosis: a literature review and update. J Clin Med..

[18] Skaug B, Assassi S (2019). Biomarkers in systemic sclerosis. Curr Opin Rheumatol.

[19] Matsushita T, Takehara K (2017). An update on biomarker discovery and use in systemic sclerosis. Expert Rev Mol Diagn.

[20] Liu C, Zhou X, Lu J, Zhu L, Li M (2019). Autophagy mediates 2-methoxyestradiol-inhibited scleroderma collagen synthesis and endothelial-to-mesenchymal transition induced by hypoxia. Rheumatology (Oxford).

[21] Raschi E, Privitera D, Bodio C, Lonati PA, Borghi MO, Ingegnoli F, Meroni PL, Chighizola CB (2020). Scleroderma-specific autoantibodies embedded in immune complexes mediate endothelial damage: an early event in the pathogenesis of systemic sclerosis. Arthritis Res Ther.

[22] Zeng W, Sun Z, Ma T, Song X, Li S, Zhang Q, Yuan W, Li J, Liu L, Zhu M (2021). Elevated ZIPK is required for TNF-alpha-induced cell adhesion molecule expression and leucocyte adhesion in endothelial cells. Acta Biochim Biophys Sin (Shanghai).

[23] La Camera G, Gelsomino L, Malivindi R, Barone I, Panza S, De Rose D, Giordano F, D'Esposito V, Formisano P, Bonofiglio D (2021). Adipocyte-derived extracellular vesicles promote breast cancer cell malignancy through HIF-1alpha activity. Cancer Lett.

[24] Wei H, Xu Y, Chen Q, Chen H, Zhu X, Li Y (2020). Mesenchymal stem cell-derived exosomal miR-223 regulates neuronal cell apoptosis. Cell Death Dis.

[25] Cammarata PR, Neelam S, Brooks MM (2015). Inhibition of hypoxia inducible factor-1alpha downregulates the expression of epithelial to mesenchymal transition early marker proteins without undermining cell survival in hypoxic lens epithelial cells. Mol Vis.

[26] Chora I, Guiducci S, Manetti M, Romano E, Mazzotta C, Bellando-Randone S, Ibba-Manneschi L, Matucci-Cerinic M, Soares R (2015). Vascular biomarkers and correlation with peripheral vasculopathy in systemic sclerosis. Autoimmun Rev.

[27] Asano Y, Sato S (2015). Vasculopathy in scleroderma. Semin Immunopathol.

[28] Chora I, Romano E, Manetti M, Mazzotta C, Costa R, Machado V, Cortez A, Bruni C, Lepri G, Guiducci S (2017). Evidence for a Derangement of the Microvascular System in Patients with a Very Early Diagnosis of Systemic Sclerosis. J Rheumatol.

[29] Tsai SH, Huang PH, Tsai HY, Hsu YJ, Chen YW, Wang JC, Chen YH, Lin SJ (2019). Roles of the hypoximir microRNA-424/322 in acute hypoxia and hypoxia-induced pulmonary vascular leakage. FASEB J.

[30] Delle Sedie A, Riente L, Maggiorini L, Pratesi F, Tavoni A, Migliorini P, Puxeddu I (2018). Potential biomarkers in patients with systemic sclerosis. Int J Rheum Dis.

[31] Cossu M, Andracco R, Santaniello A, Marchini M, Severino A, Caronni M, Radstake T, Beretta L (2016). Serum levels of vascular dysfunction markers reflect disease severity and stage in systemic sclerosis patients. Rheumatology (Oxford).

[32] Kuryliszyn-Moskal A, Klimiuk PA, Sierakowski S (2005). Soluble adhesion molecules (sVCAM-1, sE-selectin), vascular endothelial growth factor (VEGF) and endothelin-1 in patients with systemic sclerosis: relationship to organ systemic involvement. Clin Rheumatol.

[33] Zhao M, Wang S, Zuo A, Zhang J, Wen W, Jiang W, Chen H, Liang D, Sun J, Wang M (2021). HIF-1alpha/JMJD1A signaling regulates inflammation and oxidative stress following hyperglycemia and hypoxia-induced vascular cell injury. Cell Mol Biol Lett.

[34] Wipff J, Dieude P, Avouac J, Tiev K, Hachulla E, Granel B, Diot E, Sibilia J, Mouthon L, Meyer O (2009). Association of hypoxia-inducible factor 1A (HIF1A) gene polymorphisms with systemic sclerosis in a French European Caucasian population. Scand J Rheumatol.

[35] Distler JH, Jungel A, Pileckyte M, Zwerina J, Michel BA, Gay RE, Kowal-Bielecka O, Matucci-Cerinic M, Schett G, Marti HH (2007). Hypoxia-induced increase in the production of extracellular matrix proteins in systemic sclerosis. Arthritis Rheum.

[36] Takagi K, Kawamoto M, Higuchi T, Tochimoto A, Harigai M, Kawaguchi Y (2020). Single nucleotide polymorphisms of the HIF1A gene are associated with susceptibility to pulmonary arterial hypertension in systemic sclerosis and contribute to SSc-PAH disease severity. Int J Rheum Dis.

[37] Truchetet ME, Brembilla NC, Chizzolini C. Current concepts on the pathogenesis of systemic sclerosis. Clin Rev Allergy Immunol. 2021. 10.1007/s12016-021-08889-8. 10.1007/s12016-021-08889-8 PMC10167130 34487318

[38] Ciechomska M, Skalska U (2018). Targeting interferons as a strategy for systemic sclerosis treatment. Immunol Lett.

[39] Zhu J, Wang H, Zhang X, Xie Y (2017). Regulation of angiogenic behaviors by oxytocin receptor through Gli1-indcued transcription of HIF-1alpha in human umbilical vein endothelial cells. Biomed Pharmacother.

[40] Sun H, Zhang H, Li K, Wu H, Zhan X, Fang F, Qin Y, Wei Y (2019). ESM-1 promotes adhesion between monocytes and endothelial cells under intermittent hypoxia. J Cell Physiol.

[41] Licht AH, Muller-Holtkamp F, Flamme I, Breier G (2006). Inhibition of hypoxia-inducible factor activity in endothelial cells disrupts embryonic cardiovascular development. Blood.

